# Computed Tomography as a Method for Age Determination of Carnivora and Odontocetes with Validation from Individuals with Known Age

**DOI:** 10.3390/ani13111783

**Published:** 2023-05-27

**Authors:** Sina Baier-Stegmaier, Carsten Gundlach, Mariann Chriél, Mette Sif Hansen, Christina Vedel-Smith, Charlotte Vikkelsø Hansen, Daniel Klingberg Johansson, Louise Birgitte Henriksen, Magnus Wahlberg, Charlotte Bie Thøstesen, Aage Kristian Olsen Alstrup, Kristian Murphy Gregersen, Cino Pertoldi, Sussie Pagh

**Affiliations:** 1 Department of Physics, Technical University of Denmark, 2800 Kongens Lyngby, Denmark; sbaier@fysik.dtu.dk (S.B.-S.);; 2 Department of Health Technology Center for Diagnostics, Technical University of Denmark, 2800 Kongens Lyngby, Denmark; 3 Department of Veterinary and Animal Sciences, University of Copenhagen, 1870 Frederiksberg C, Denmark; 4 Department of Research and Collections, Natural History Museum Aarhus, 8000 Aarhus, Denmark; cvs@nathist.dk (C.V.-S.);; 5 Natural History Museum of Denmark, University of Copenhagen, 1165 København, Denmark; 6 Department of Biology, University of Southern Denmark, 5230 Odense, Denmark; 7 Marine Biological Research Center, University of Southern Denmark, 5300 Kerteminde, Denmark; 8 The Fisheries and Maritime Museum, 6710 Esbjerg, Denmark; 9 Department of Clinical Medicine, Aarhus University, 8200 Aarhus, Denmark; 10 Department for Nuclear Medicine & PET, Aarhus University Hospital, 8200 Aarhus, Denmark; 11 Institute of Conservation, Royal Danish Academy, 1435 København, Denmark; 12 Department of Chemistry and Bioscience—Section of Biology and Environmental Science, Aalborg University, 9220 Aalborg, Denmark

**Keywords:** museum collections, X-ray, computed tomography, micro-CT, µ-CT, toothed whale age, carnivore age, red fox, *Vulpes vulpes*, American mink, *Neogale vison*, harbor porpoise, *Phocoena phocoena*

## Abstract

**Simple Summary:**

Especially when it comes to ancient and valuable museum samples of rare or extinct species, non-invasive methods for aging specimens are preferable. X-ray micro-computed tomography (µ-CT) is considered a non-invasive technique for age determination of mammalian carnivores and toothed whales. Teeth from 13 red foxes (*Vulpes vulpes*), 2 American mink (*Neogale vison*), and 2 harbor porpoises (*Phocoena phocoena*) of known age were examined using µ-CT. The number of visible dental growth layers extracted from the µ-CT data was highly correlated with the true age of the individuals for all three species.

**Abstract:**

Traditional methods for age determination of wildlife include either slicing thin sections off or grinding a tooth, both of which are laborious and invasive. Especially when it comes to ancient and valuable museum samples of rare or extinct species, non-invasive methods are preferable. In this study, X-ray micro-computed tomography (µ-CT) was verified as an alternative non-invasive method for age determination of three species within the order of Carnivora and suborders Odontoceti. Teeth from 13 red foxes (*Vulpes vulpes*), 2 American mink (*Neogale vison*), and 2 harbor porpoises (*Phocoena phocoena*) of known age were studied using µ-CT. The number of visible dental growth layers in the µ-CT were highly correlated with true age for all three species (R^2^ = 96%, *p* < 0.001). In addition, the Bland–Altman plot showed high agreement between the age of individuals and visible dental layers represented in 2D slices of the 3D µ-CT images. The true age of individuals was on average 0.3 (±0.6 SD) years higher than the age interpreted by the µ-CT image, and there was a 95% agreement between the true age and the age interpreted from visible dental layers in the µ-CT.

## 1. Introduction

Age determination is crucial for population studies (e.g., demography, life tables, mortality, age-related reproduction, cause of death, body size, and asexual dimorphism) [1,2,3,4,5,6,7,8,9,10]. Several methods have been explored to determine the age of mammalian carnivores and toothed whales (e.g., cranial dimensions, dry weight of eye lenses, degree of fusion of cranial sutures, occlusion of the pulp cavity, or increments in dentine layers) [11,12,13,14,15,16,17,18]. With such methods, juveniles can be separated from adults, but they can only provide an approximate age of the individual.

Dental layer counting is widely accepted as the standard method for determining the age of mammalian carnivores and toothed whales [11,19,20]. The development of a pulp cavity (which is wider in young animals) and dentine layers are species-specific. In carnivores and toothed whales, one dentine layer is normally formed each year [12,21,22]. In general, the cementum lines in individuals from the families Canidae and Mustelidae are found in the tooth root outermost from the neonatal line (the line separating the primary and secondary dental layer) where each line represents one year [12,21]. In contrast, dental growth layer groups (GLGs; several thin groups of layers) in toothed whales are counted in the dentine crown on the inside of the neonatal line [17,18,22].

For mammalian carnivores and toothed whales, the tooth-preparation methods vary to some extent. In the most often used treatment, a tooth is decalcified for 2–48 h, after which it is sliced in thin sections of 20–30 μm with a microtome, stained with tissue color, and mounted on object glass before the age lines are visible and can be counted [1,11,17,18,19,20,23]. Alternatively, teeth are ground roughly to half their thickness using sandpaper and then smoothened and finally polished [20,24].

Once the dental layers are visible, they can be counted under a stereo microscope (sometimes iteratively because some layers may be more visible at different focal depths). Although this is a common procedure for determining the age of mammal carnivores and toothed whales, the process is to some extent prone to errors due to subjectivity of the person performing the work [21]. Most of all, in all the traditional methods it is necessary to split the tooth into half to determine the age of the individual. This is especially challenging when studying valuable museum collections of rare or extinct species or fossils of younger age. An example of the potential use of some features in fossil carnivoran teeth was recently mentioned [25].

An alternative and non-invasive technique is X-ray micro-computed tomography (µ-CT). µ-CT scans allow us to obtain a full 3D image of the studied object, which can be used to digitally preserve the object, virtually cut the object at different angles and study the resulting 2D slices, and measure various desired parameters. The contrast is based on different X-ray absorption properties of the material depending on differences in the density and atomic number of the investigated material. Thus, a material with a higher atomic number or density appears brighter in the final 3D image than a material with a lower atomic number or density. Earlier attempts at age determination with structurally non-invasive techniques such as magnetic resonance imaging (MRI) and X-ray CT have not been successful [26,27]. However, Velasco et al. [28] used synchrotron-based X-ray CT for successful visualization of dental lines in the cementum of three species of African bovids.

The aim of this study was to test X-ray µ-CT as a tool to read the dental lines of animals from two species within the order Carnivora and one species within the suborder Odontoceti and to compare layers found in the dental cementum with the known age of the individuals.

## 2. Materials and Methods

X-ray µ-CT was conducted on teeth and roots from individuals of known age of the three species: red foxes (*Vulpes vulpes*) (*n* = 13), American mink (*Neogale vison*) (*n* = 2), and harbor porpoises (*Phocoena phocoena*) (*n* = 2). We used the canine teeth of mink and foxes (largest teeth) because they were the easiest to handle. Porpoise teeth are all alike in their shape and size (homodont). For further analysis and visualization, the software “Avizo Lite 2020.20” (Thermo Fisher Scientific, Waltham, MA, USA) was used. Hereafter, red fox, American mink, and harbor porpoise are referred to as fox, mink and porpoise.

The teeth of 13 fox skeletons of known age were stored at The Natural History Museum of Aarhus (NHM 13,000 to 13,202 from the museum’s collection) for around 50 years. The foxes had been ear-tagged as cubs from 1965 to 1971 and recovered when shot or found dead in 1966 to 1976 [29]. Therefore, the exact ages of these foxes were known. In foxes, the first cementum line develops between May and August of the fox’s second year of life, after which a line develops annually in the autumn [11,12]. The cementum layers appear as characteristic rings consisting of paler opaque (summer) and darker transparent (winter) areas when using a stereo microscope [12].

To test the effect of the length of storage on ability to visualize dental lines with µ-CT, a tooth of one fox that had been killed in 2012 and prepared in 2015 (according to Roulichová et al., 2007 [20]) was analyzed. The tooth had been dried and stored at room temperature for three years before conducting the µ-CT. Between 2012 and 2015, the fox tooth was deep frozen. The age of this fox was estimated at 4 years using the traditional method [20].

Two mink (both at the age of two years) were sampled from Danish mink farms in 2018. In mink and other members of the family Mustelidae, cementum layers are difficult to discriminate via traditional methods. In many individuals assessed as adults, no incremental lines can be observed [2,30]. Therefore, Eurasian pine martens (*Martes martes*) and mink (and perhaps also for other mustelids), animals are aged by using a model describing the relationship between the pulp cavity width and the age of the individual [2,16].

The two harbor porpoises (named Frigg (harbor porpoise 1) and Sif (harbor porpoise 2)) were born or kept in captivity since they were juveniles at Fjord&Bælt, an aquarium and research institution in Denmark. Their ages were thereby known to be 5.5 and 13.5 years, respectively [31]. In harbor porpoises, GLGs develop on the inside of the neonatal line in the dentine crown that are visible as dark lines or “arcs” formed annually between January and September [12,17].

### 2.1. µ-CT of Foxes and Porpoises

The µ-CT of fox and porpoise teeth fixed on the sample holder in a floral sponge was obtained using a “Nikon XT H 225 ST” (Nikon Metrology Europe NV Leuven, Belgium) metrology device. The instrument was operated with a tungsten reflection target using 55 kV and 15 W. For image acquisition, 1571 projections with 1 s exposure time and a 2 × 2 binning were used, and 8 frames were averaged per projection. A total acquisition time of 4 h and 21 min was achieved per sample. Depending on the sample, the reconstructed isotropic voxel size was approximately 15 µm (Table 1). The reconstruction of the tomographs was performed using the software “CT Pro 3D” (Nikon Metrology Europe NV Leuven, Belgium)) and included a correction for beam hardening effects.

### 2.2. µ-CT of Mink

The µ-CT of the mink teeth (all fixed on the sample holder with reusable adhesive gum), were obtained using a “ZEISS Xradia 410 Versa” (Carl Zeiss Microscopy, Oberkochen, Germany) device. The instrument was operated with a tungsten reflection target using 50 kV and 10 W, a ZEISS “low energy one” (LE1) filter, and a large field of view (LFOV) objective. A total of 1601 projections with 5 s exposure time per projection were recorded, and an isotropic voxel size of 15.8 µm was obtained after a 2 × 2 binning. The full acquisition time was 3 h and 8 min in total. Image reconstruction was performed using the inbuilt acquisition and reconstruction software package (version 14.0.14829.38124) provided by ZEISS based on an algorithm using filtered back-projection [32].

### 2.3. Statistical Analysis

The true ages of the individuals of the three species (fox, mink, and harbor porpoise) were compared with counts of dentine layers of representative 2D slices of the 3D CT images using a linear regression analysis (Pearson product–moment correlation test) and a Bland–Altman plot.

## 3. Results

The teeth from 13 red foxes with known age, 2 mink, and 2 porpoises were all analyzed using µ-CT.

**Figure 1:**
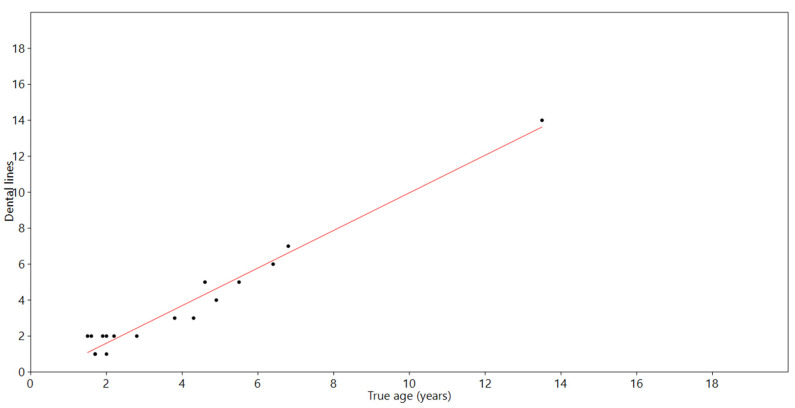
Linear regression between true age of all species and number of dental layers visible in CT-based measurements (R^2^ = 96%, *p* < 0.001).

**Figure 2:**
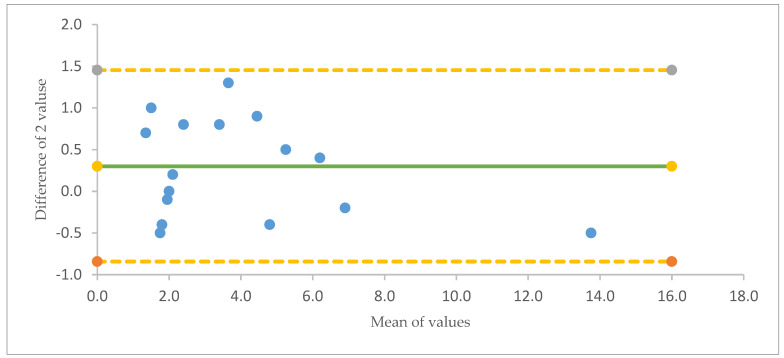
Bland–Altman Plot of true age (years) of individuals vs. the number of dental layers visible in a 2D slice of the 3D CT images. On average (green line), individuals were 0.3 years older than the number of lines visible. There was a 95% match in age between the real age and the age interpreted from dental layers. The number of lines visible were expected to fall in the range of -0.84 years (yellow line) and +1.45 years (yellow line) around the true age.

**Figure A1:**
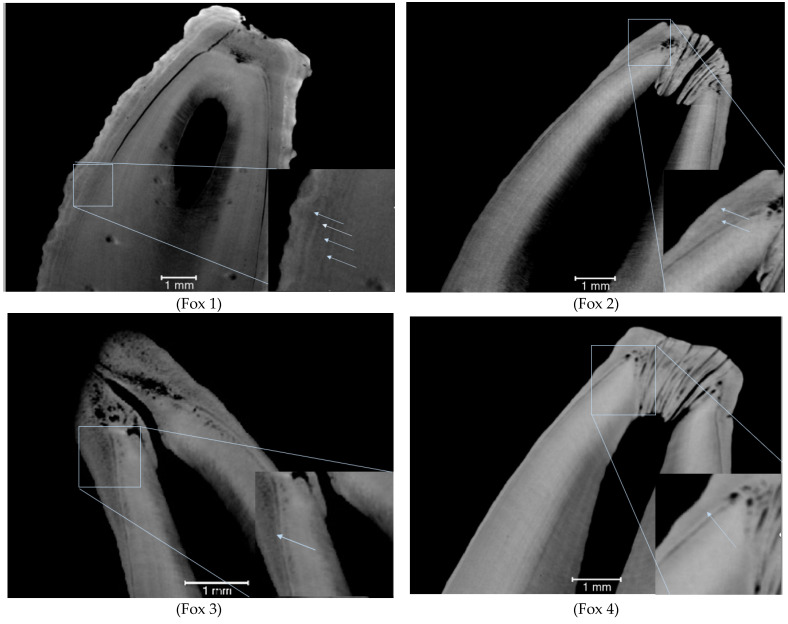

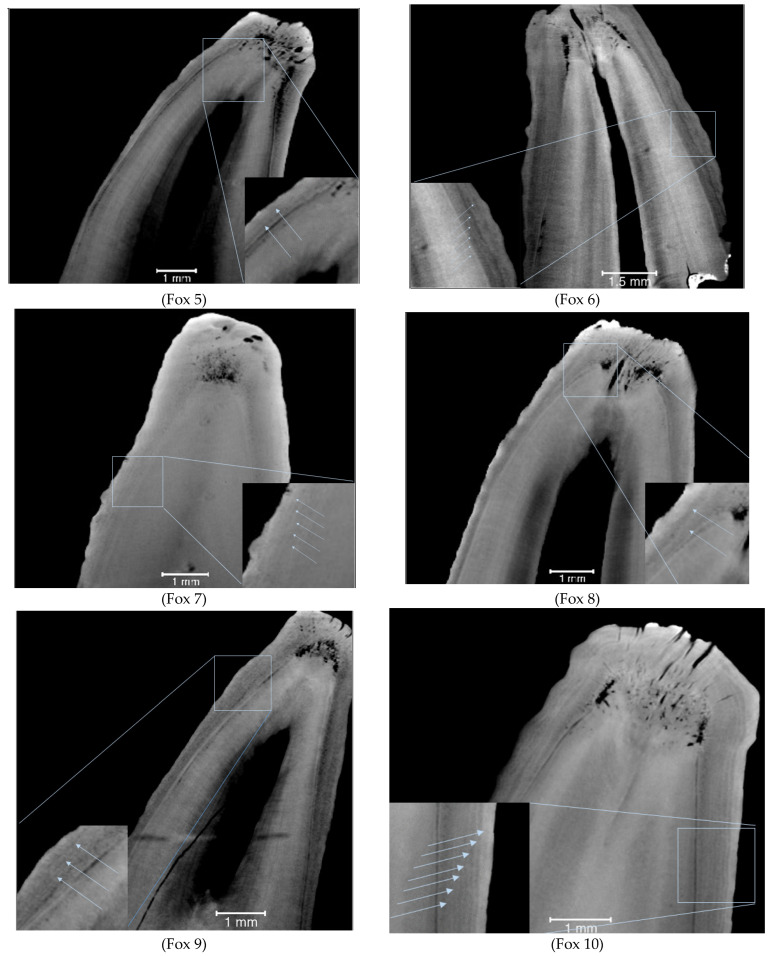

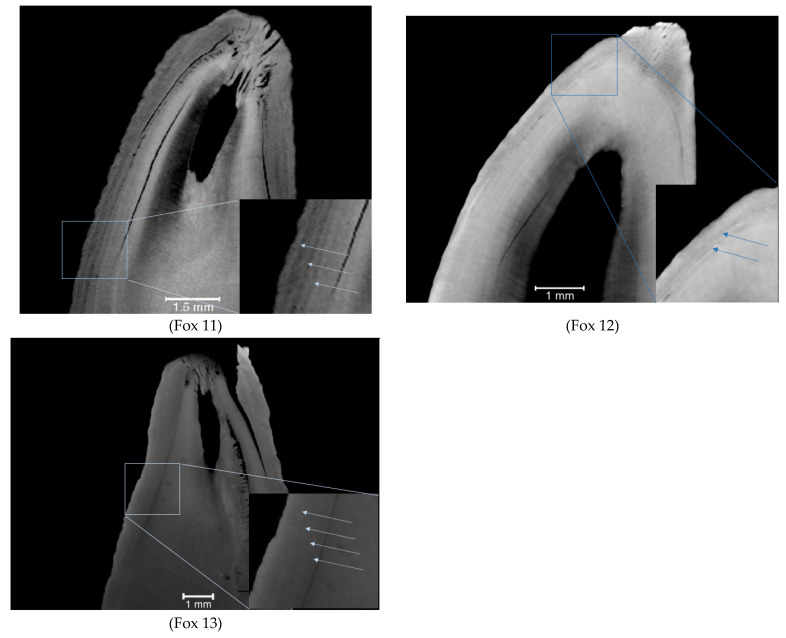
Fox 1–Fox 13. Visualizations of 2D slices of the 3D CT images of the 13 foxes in Table 2.

**Figure A2:**
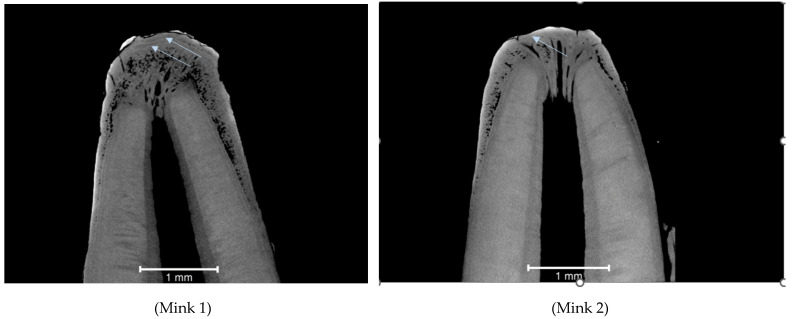
Mink 1 and Mink 2. Visualizations of 2D slices of the 3D CT image the two mink in Table 2.

**Figure A3:**
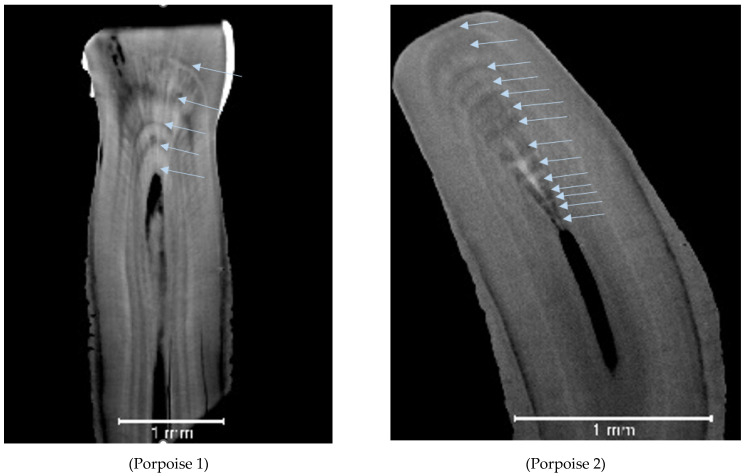
Porpoise 1 and Porpoise 2. Visualizations of 2D slices of the 3D CT image of the two porpoises in Table 2.

There was a high agreement between dental layers visualized using CT and the real age of the individuals (Table 2; Appendix A Figure A1, Figure A2 and Figure A3); the number of layers and GLGs visible in the teeth were highly correlated with the exact ages of the foxes, mink, and harbor porpoises (R^2^ = 96%, *p* < 0.001), and the Bland–Altman plot indicated low variation and similar deviations when comparing the age of the individual and dental layers visible in the CT measurement (Figure 1 and Figure 2). The average difference was −0.3 years; i.e., the true age was slightly higher than the estimated one, and the 95% confidence interval was −0.82 years to +1.45 years.

**Figure 3:**
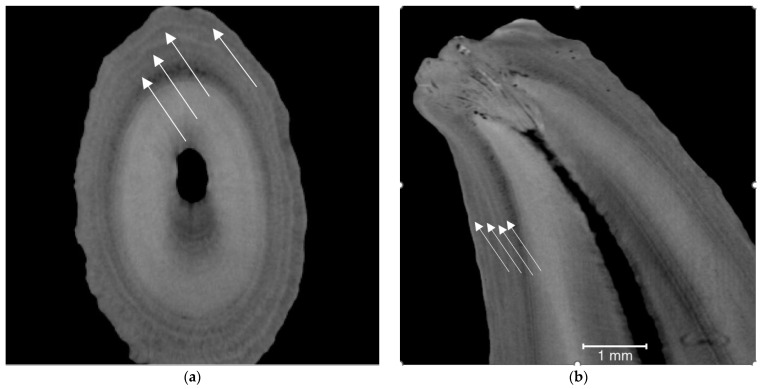
Two-dimensional images of fox tooth stored at room temperature for only 3 years before µ-CT: (**a**) transverse; (**b**) longitudinal. The fox was estimated to be 4 years old. Arrows point to dark layers.

For the additional fox tooth that had been stored at room temperature for 3 years and was estimated to be 4 years old by conventional methods, four clearly visible dental layers were observed via µ-CT; these lines were much clearer than the lines in teeth of specimens stored dry and at room temperature for around nearly 50 years in a museum’s collection (Figure 3). A movie showing fox R0182 in 3D, and during virtual slicing, can be found in the supplementary materials (S1).

## 4. Discussion

Despite minor variations in image quality, there was a high agreement between the known age and the number of dental lines visible in the dentine layer (Table 2; Figure 1 and Figure 2; Appendix A Figure A1, Figure A2 and Figure A3). For a 95% confidence interval, one would expect a maximum difference between the interpretation by visible lines in the CT image and true age to be 1.5, whereas the biggest observed maximum was found to be 1.3 (see the Bland–Altman plot in Figure 2). In comparison, in a previous study of the age of Danish red foxes with known age and aged using the traditional method, the age estimation was correct in 93% (125 of 135) of the cases [12].

In carnivores, dental lines or layers are normally read outside the neonatal line, whereas in toothed whales the GLGs are read inside the neonatal line. However, in both carnivores and toothed whales, dental layers could be recognized on both sides of the neonatal line when examining the µ-CT images. For one mink, where two lines would be expected (Appendix A Figure A2 (Journal No. 15700_1)), only one line was visible on the outside of the neonatal line, while both lines were easily visible on the inside of the neonatal line. However, with careful examination, lines may be recognized on both sides of the neonatal line, but it was still our experience that it was easiest to read lines outside the neonatal line in the root of carnivores and in the dentine in toothed whales.

The different quality of CT images of dental layers—some had well-defined layers and some had blurred or faint layers—may have relied on different qualities of the cadavers before the skeletons are preserved as well as the health and growth patterns of the individuals. The time of storage may also have influenced the visibility of the dental lines in the foxes. Dental lines in a tooth from a fox stored for only 3 years were much clearer than those in teeth from foxes stored for around 50 years (Figure 3 vs. Appendix A Figure A1, Figure A2 and Figure A3). However, this assumption was based on a single sample, which does not allow us to draw strong conclusions; therefore, it is recommended to carry out further research in which the objective is to know the effect of storage with a larger sample size.

Previously, µ-CT studies using clinical scanners of sperm whale (*Physeter macrocephalus*) teeth have failed to visualize dental layers [26,27]. The present study showed that µ-CT was not only able to visualize growth layers in teeth of carnivores and toothed whales but also could do so to an extent that they could be aged to a quality comparable to that of traditional methods.

## 5. Conclusions

To our knowledge, this was the first successful age determination of carnivores and toothed whales based on structurally non-invasive teeth X-ray CT.

The present study showed that µ-CT was not only able to visualize growth layers in the teeth of carnivores and toothed whales but also could do so to an extent that they could be aged to a quality comparable to that of traditional methods. The use of µ-CT does not imply the same destructive and time-consuming sample preparation, which is especially a benefit when imaging rare specimens for which preservation is required. If several teeth are imaged at the same time, the technique also outcompetes fast traditional preparation techniques. Furthermore, using µ-CT has a huge potential for extended information based on quantitative image analysis of the contrast differences in and between growth layers, which might provide information on feed availability and pregnancies in addition to (semi)-automized age determination.

## Figures and Tables

**Table 1:** Isotropic reconstructed voxel size used in the different measurements of foxes and harbor porpoises.

Journal Number	Isotropic Voxel Size/µm
Fox 1 (838–839)	14.6
Fox 2 (948–949)	14.4
Fox 3 (1051–1052)	12.9
Fox 4 (3113–3114)	13.9
Fox 5 (3215–3216)	14.7
Fox 6 (894–895)	18.2
Fox 7 (689–690)	11.0
Fox 8 (707–708)	14.7
Fox 9 (3420–3421)	12.9
Fox 10 (741–742)	12.4
Fox 11 (4505–4506)	146
Fox 12 (705–706)	14.7
Fox 13 (693–694)	14.7
Harbor porpoise 1	6.4
Harbor porpoise 2	3.7
Fox R0182	14.5

**Table 2:** Comparison of known age with the number layers visible in representative 2D slice of the 3D CT image.

Species No. and Journal No.	Age of Individual (Years)	Number of Dental Layers
Fox 1 (838–839)	4.9	4
Fox 2 (948–949)	1.5	2
Fox 3 (1051–1052)	1.7	1
Fox 4 (3113–3114)	1.7	1
Fox 5 (3215–3216)	1.9	2
Fox 6 (894–895)	6.4	6
Fox 7 (689–690)	4.6	5
Fox 8 (707–708)	2.8	2
Fox 9 (3420–3421)	2.2	3
Fox 10 (741–742)	6.8	7
Fox 11 (4505–4506)	4.3	3
Fox 12 (705–706)	1.6	2
Fox 13 (693–694)	3.8	3
Mink 1	2	2
Mink 2	2	1
Harbor porpoise 1	5.5	5
Harbor porpoise 2	13.5	14

## Data Availability

The data presented in this study are available upon request from the corresponding author.

## Supplementary Materials

The following supporting information can be downloaded at: https://www.mdpi.com/article/10.3390/ani13111783/s1, Video S1: Movie showing fox R0182 in 3D and during virtual slicing. Some ring artefacts, arising from the image acquisition, appear as bright and dark spots or rings, dependent on the direction used for virtual slicing
